# Genetic Diversity and Hybridisation between Native and Introduced Salmonidae Fishes in a Swedish Alpine Lake

**DOI:** 10.1371/journal.pone.0152732

**Published:** 2016-03-31

**Authors:** Leanne Faulks, Örjan Östman

**Affiliations:** 1 Department of Ecology and Genetics – Animal Ecology, Uppsala University, Norbyvägen 18D, 75236 Uppsala, Sweden; 2 Sugadaira Montane Research Centre, University of Tsukuba, Sugadaira-kogen 1278–294, Ueda, Nagano, 386–2204, Japan; 3 Department of Aquatic Resources, Institute of Coastal Research, Skolvägen 6, 742 42 Öregrund, Sweden; University of Iceland, ICELAND

## Abstract

Understanding the processes underlying diversification can aid in formulating appropriate conservation management plans that help maintain the evolutionary potential of taxa, particularly under human-induced activities and climate change. Here we assessed the microsatellite genetic diversity and structure of three salmonid species, two native (Arctic charr, *Salvelinus alpinus* and brown trout, *Salmo trutta*) and one introduced (brook charr, *Salvelinus fontinalis*), from an alpine lake in sub-arctic Sweden, Lake Ånn. The genetic diversity of the three species was similar and sufficiently high from a conservation genetics perspective: corrected total heterozygosity, H’_T_ = 0.54, 0.66, 0.60 and allelic richness, A_R_ = 4.93, 5.53 and 5.26 for Arctic charr, brown trout and brook charr, respectively. There were indications of elevated inbreeding coefficients in brown trout (G_IS_ = 0.144) and brook charr (G_IS_ = 0.129) although sibling relationships were likely a confounding factor, as a high proportion of siblings were observed in all species within and among sampling locations. Overall genetic structure differed between species, Fst = 0.01, 0.02 and 0.04 in Arctic charr, brown trout and brook charr respectively, and there was differentiation at only a few specific locations. There was clear evidence of hybridisation between the native Arctic charr and the introduced brook charr, with 6% of individuals being hybrids, all of which were sampled in tributary streams. The ecological and evolutionary consequences of the observed hybridisation are priorities for further research and the conservation of the evolutionary potential of native salmonid species.

## Introduction

The conservation and maintenance of biodiversity is important for the stability and functioning of ecological communities and populations and also provides immeasurable benefits to human society such as ecosystem services and aesthetic value [1]. Biodiversity can be assessed at various hierarchical levels, from the ecosystem all the way down to genetic diversity [2]. From a conservation management perspective it is most often the species that is the unit of consideration [3], yet species are classified by the human mind as an attempt to make order of the natural world. In reality, the natural world is not fixed but is changing, thus species exist on a continuous spectrum that evolves in space and time. This can pose challenges for conservation, as situations may arise where management of the ‘species’ is inappropriate [3], e.g. cryptic species [4], recent/ongoing diversification [5] and hybridisation [6].

Diversification (or speciation) research has traditionally focused on the level of species and above, but diversification below this level is of equal importance and interest [7, 8]. The diversification of taxa is promoted by a variety of selection pressures, such as variation in environmental conditions and competition for mates and/or resources, and diversification processes occur across a wide range of temporal and spatial scales ([9], for a specific review of fishes see [10]), for example, the rapid adaptive radiation of cichlids within a single lake (e.g. [11]), the distribution of a single species lineage across a large area (e.g. whale sharks, [12]), and ‘old species’, which remain relatively unchanged over evolutionary time (e.g. lungfish, [13]). Understanding the processes underlying diversification will aid in formulating appropriate conservation management plans that help maintain the evolutionary potential of taxa, particularly under human-induced activities and climate change [14–17].

Hybridisation is defined as the interbreeding of two different species and can have a variety of outcomes, including promoting speciation [18], introgression and hybrid swarms [19], hybrid vigour and adaptation [20], and reverse speciation [21, 22]. As such hybridisation can be viewed as a significant evolutionary process [23, 24]. Judgement of whether the outcomes of hybridisation have positive or negative consequences has often been dependent on the taxa studied (e.g. plants vs animals). Of greater relevance for conservation management purposes is whether the underlying cause of hybridisation is natural or anthropogenic. Natural hybridisation (among native species in their native range without anthropogenic influences) is observed in several taxa, particularly plants [25] and fishes [26, 27]. However, there is increasing concern over the role of anthropogenic hybridisation in shaping contemporary biodiversity. Hybridisation between native and introduced species is one obvious example [28], but there are also reports of hybridisation being induced between two native species due to habitat destruction and climate change [29].

Freshwater fishes have one of the highest frequencies of both recent adaptive radiations and hybridisation among all animal taxa [27]. The restricted nature of freshwater environments—freshwaters constitute just 0.8% of the earth’s surface, yet are host to 6% species’ diversity [30]—is thought to be one of the main contributing factors [27]. Freshwater ecosystems are also one of the most threatened in the world [31], being impacted by invasive species [32], habitat destruction and fragmentation [33], water abstraction [34], fisheries activities [35], aquaculture [36] and climate change [37]. Polar and alpine regions may be particularly sensitive as their biota have limited options for migrating to more suitable conditions [38, 39]. One group of fishes distributed in these regions and which also exhibits high levels of diversification and hybridisation is the Salmonidae [27].

Diversification in postglacial fishes, especially salmonids, is a widely recognised phenomenon, with variation occurring not only across a species range, but also within individual lakes. The salmonid family is composed of over 60 species, many of which include different ecotypes or spatially subdivided morphs [40]. For example, the genera *Salvelinus* and *Salmo* provide some classic cases of differential diets [41, 42], phenotypic and morphological variation [43, 44], as well as genetic divergence [45, 46]. These local adaptations are mainly driven by niche partitioning and resource specialisation to reduce intra-specific competition, and may be reinforced by spatial or temporal spawning site differentiation and specific mate choices [47, 48]. These studies of diversification and hybridisation have mostly investigated areas within the species historical and recent natural distributions [49–52].

However, due to their popularity as recreational fishing targets and their extensive and widespread translocation, it is important to consider processes of diversification and hybridisation in locations where both native and introduced salmonid species are present. This study assessed the genetic diversity and structure of three salmonid species, two native (Arctic charr, *Salvelinus alpinus* and brown trout, *Salmo trutta*) and one introduced (brook charr, *Salvelinus fontinalis*), from an alpine lake in sub-arctic Sweden, Lake Ånn. The overall aims were to: 1) determine levels of genetic diversity and compare to previous studies of these species; 2) determine the extent of genetic diversification and structure in relation to geographic location, including recognised spawning locations; and 3) detect if any hybridisation is occurring, particularly between the native and introduced charr (*Salvelinus*) species.

## Methods

### Study species and location

**Fig 1:**
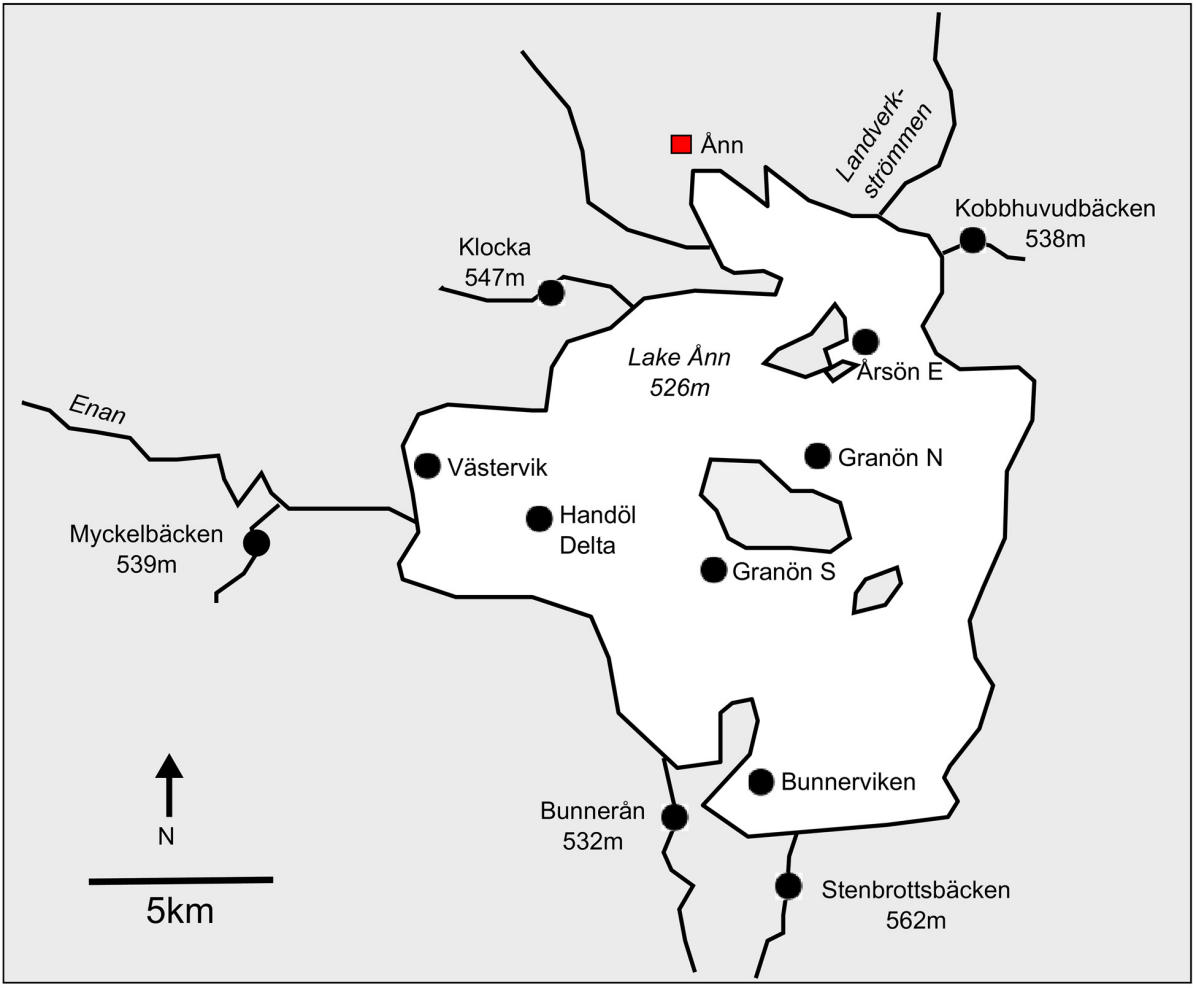
Map showing the study area in and around Lake Ånn. Sampling locations are indicated with black circles and labelled with the location name and altitude (m).

Lake Ånn is situated in central Sweden (63.261212°N, 12.567719°E) at an elevation of 526m (Fig 1). The lake is approximately 57 km^2^ in area and has one main tributary, Enan, and one main downstream outflow, Landverksströmmen. The lake ranges in depth from 1–39.5m with the majority of the lake being less than 2 m deep (e.g. around the Handöl Delta), and areas deeper than 10m being restricted to the south-western area [53]. Barriers to dispersal such as weirs and waterfalls are present in the majority of tributaries. Three native fish species, brown trout, *Salmo trutta*, Arctic charr, *Salvelinus alpinus*, and Eurasian minnow, *Phoxinus phoxinus*; and one introduced species lake trout, *Salvelinus namaycush*, are known to inhabit the lake [53]. The introduced brook charr, *Salvelinus fontinalis*, is also present in the tributaries [53]. Lake trout were first recorded in Lake Ånn in 1974, but since efforts to remove the species began in the 1990s the population size is now thought to be minimal and under control [53]. However, there is no active effort to remove the introduced brook charr. Details of the introduction of brook charr are unclear. The species was first introduced to Sweden from North America in the 1850s [54] as a recreational fishing target, but its specific history in Lake Ånn is unknown. In addition, there are no records of stocking of the native salmonids in the Lake Ånn area. Historically, local fisherman recognised specific spawning locations and times of each species across the lake [53]. For example, Arctic charr spawned in early autumn in the lake around Granön N, Granön S, and Årsön E and in late autumn around Bunnerviken. Brown trout spawned in early autumn in a few larger tributaries including Herrån. Arctic charr have also been observed using some streams as spawning grounds, e.g. Bunnerån and Enan [53].

### Sampling, DNA extraction, sequencing and genotyping

Arctic charr, brown trout and brook charr were sampled by local licensed fisherman from Lake Ånn using gillnets from July to November 2012, and by the authors from the tributaries surrounding the lake by electrofishing in September 2012 (Fig 1). Each tributary was sampled over approximately 100–200 metres to try to reduce sampling of local family groups [55]. The sampling times spanned over the spawning period of all three species, and may include spawning aggregations and/or foraging groups. A small (max 1cm^2^) tissue biopsy was taken from the caudal fin of each fish using sterile scissors. When possible fish were returned the water alive. Whilst electrofishing, individuals were identified to genus level only, i.e. *Salmo* or *Salvelinus*, as distinguishing between young (most fish were less than 50mm) Arctic and brook charr in the field was difficult. Tissue was frozen and transported to Uppsala University for analysis.

DNA was extracted from tissue samples using a modified salting out protocol [56]. In order to identify charr individuals to the species level, the mitochondrial cytochrome oxidase I (COI) region was amplified using the primers COI F: TTCTCAACTAACCAYAAAGAYATYGG and COI R: TAGACTTCTGGGTGGCCRAARAAYCA [57] and PCR conditions described in [58]. PCR products were cleaned using an EXOSAP procedure and samples were sequenced, using the forward primer. Twenty-five microsatellite loci were scanned for amplification and variation across all study species. Finally, a total of 14 loci were amplified in Arctic charr, nine in brook charr and 11 in brown trout, with four common loci among all species and eight common loci between the two charr species (see Table A in S1 File for primer details and references). All loci were multiplexed using QIAGEN TypeIt kits following the manufacturers guidelines. All PCRs had an annealing temperature of 56°C and 30 cycles. Sequencing and genotyping was performed on an ABI3730xl at Uppsala University. Sequences were aligned in MEGA 5 [59] and blasted to determine species designations. Sequences were submitted to GenBank (Acc Nos KU896910-896913 and KU933676-933677). All charr individuals were identified according to their COI barcode. All microsatellites were genotyped using GeneMarker 1.85 (SoftGenetics LLC, State College PA, USA, 2009)(S1 Tables).

### Data Analysis

Deviations from Hardy-Weinberg equilibrium and the presence of null alleles, stuttering and large allele dropout were assessed in Microchecker v2.2.3 [60]. Visual representation of the genetic differentiation among the three species (4 loci) was created using a principal components analysis performed on the entire dataset (Genodive, [61]). Following the identification of several potential charr hybrids in the PCA we used NewHybrids (8 loci, 15000 steps with Jefferson priors) to determine the probability of each individual being pure, F1, F2 or a backcross [62]. Only pure-bred individuals were included in the subsequent population genetics analyses. COLONY [63] was used to assess presence of siblings (full and half), which can be common in salmonid sampling and is known to affect inferences of genetic structure. Simulation studies of STRUCTURE analyses, with 10 and 20 loci in an initial population of 70 individuals, have shown that full sibling groups of 6 and 9 and half sibling groups of 17 and >35, can cause the inference of K = 2 [64]. COLONY analyses were run with the recommended input parameters as follows: without updating the allele frequency during the annealing process, with sibship size scaling, a single run of medium length, the full likelihood analysis method with high precision, assuming polygamous males and females and no inbreeding, allelic dropout rates of zero, and other typing error rates of 0.001. Pairs of individuals were considered siblings if the probability calculated by COLONY was greater than 0.5. When full sibling pairs were identified, one individual of the pair was removed prior to further analyses. Estimates of heterozygosity (H_O_/H_S_/H’_T_), inbreeding coefficients (G_IS_), pairwise genetic differences (F_ST_, with Bonferroni corrected significance values), and AMOVA were calculated in Genodive [61]. Allelic richness (A_R_) was calculated in FSTAT [65]. A high proportion of sibling relationships (and low Fst values) were observed in all three species (see details in Results below), suggesting any further analyses of genetic structure such as Mantel tests or STRUCTURE assignment would be unlikely to reveal meaningful insights into genetic diversification related to geographic location, due to the confounding effect of kinship structure.

## Results

### Phylogeography and hybridisation

91 brown trout and 75 Arctic charr individuals were collected from Lake Ånn, and 49 brown trout, eight Arctic charr and 96 brook charr individuals were collected from tributaries. There were two COI haplotypes in Arctic charr, which varied by a single mutation, A/G, at site 386. Although only the G haplotype was detected in Bunnerån, there was no other strong phylogeographic distribution of the COI haplotypes across the lake and its tributaries (Table 1). Only one brook charr haplotype was detected. In contrast, three brown trout haplotypes were detected, one common and two rare, which were found in single individuals from Bunnerviken and Granön N.

**Table 1:** Population genetic parameters for the fishes of Lake Ånn.

Location	N	COI (A/G)	H_O_/H_S_	A_R_	G_IS_
***Arctic charr***					
**Bunnerån**	5	0/5	0.57/0.48		-0.191
**Bunnerviken**	20	8/9	0.55/0.54	5.05	-0.028
**Granön N**	12	6/6	0.56/0.55	4.75	-0.016
**Granön S**	21	3/18	0.55/0.51	4.35	-0.085
**Handöl Delta**	22	11/9	0.58/0.58	4.69	-0.014
**Kobbhuvudb.**	3 (2)	2/1	0.64/0.64		0.000
***Brook charr***					
**Klocka**	32		0.54/0.55	4.49	0.018
**Kobbhuvudb.**	4		0.56/0.59		0.055
**Myckelb.**	20		0.45/0.53	4.55	0.145
**Stenbrottsb.**	29 (24)		0.48/0.66	5.75	0.273
***Brown trout***					
**Årsön E**	7		0.46/0.73		0.374
**Bunnerviken**	20		0.56/0.63	5.10	0.117
**Granön N**	30		0.54/0.65	5.60	0.170
**Granön S**	9		0.66/0.75		0.115
**Herrån**	14		0.42/0.54	4.27	0.223
**Klocka**	7		0.61/0.61		-0.010
**Kobbhuvudb.**	5		0.59/0.62		0.047
**Myckelb.**	20		0.54/0.65	6.07	0.174
**Västervik**	19		0.56/0.59	5.02	0.062

N = total sample size (pure individuals in brackets), COI (A/G) = the number of individuals with the A or G mtDNA COI haplotype, H_O_ = observed heterozygosity, H_S_ = expected heterozygosity within subpopulation, A_R_ = allelic richness, G_IS_ = corrected inbreeding coefficient

**Fig 2:**
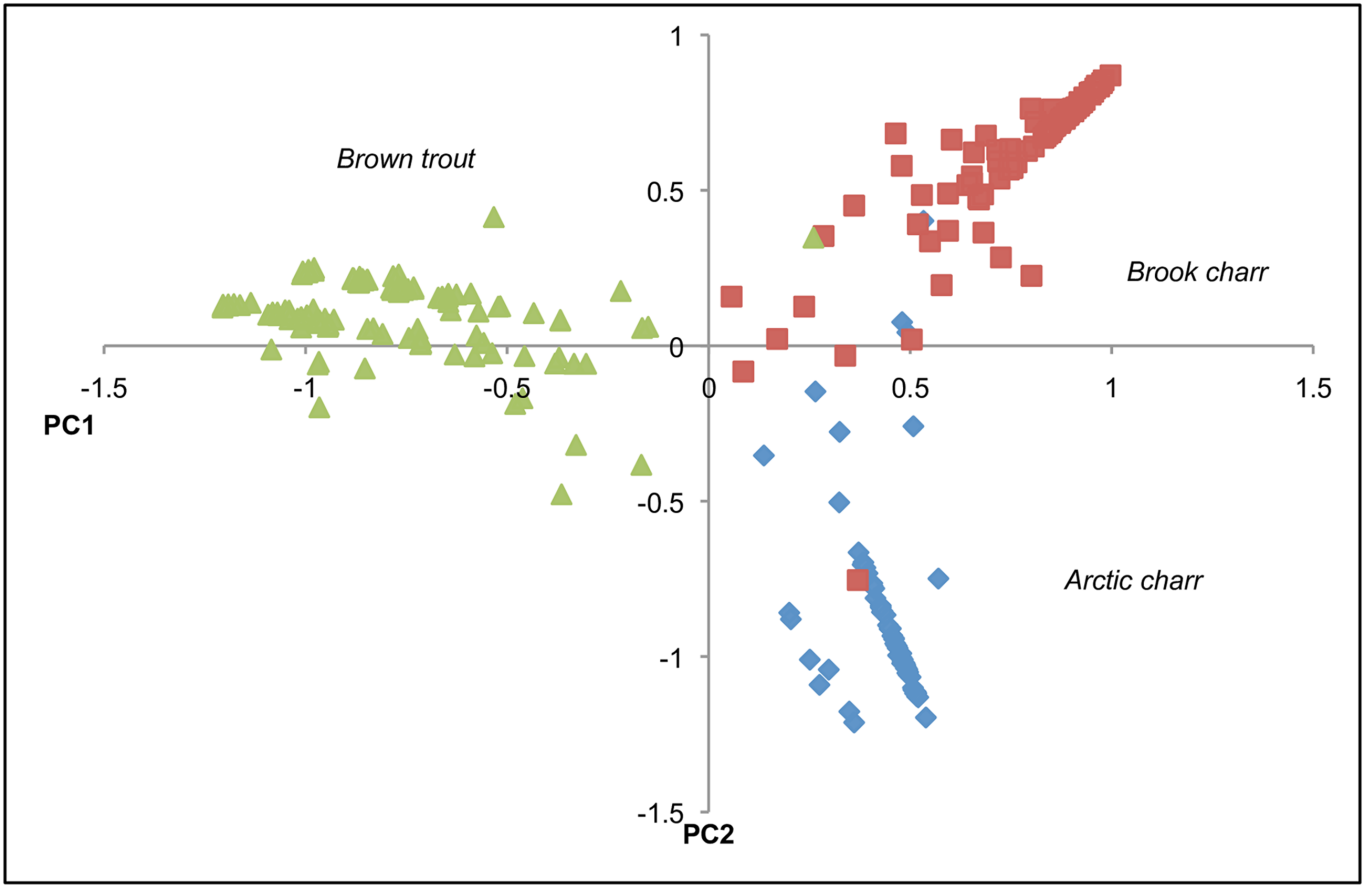
Graphical display of the first two principal components of the principal components analysis (PCA) of the microsatellite dataset of all three species, brown trout (green triangles), brook charr (red squares) and Arctic charr (blue diamonds).

Principle components analysis of microsatellites from all three species combined revealed a clear separation of brown trout from the 2 charr species along the first PC-axis (Fig 2). Although the two charr species differed along the second PC-axis, there was an area of overlap between them (Fig 2). Further analysis of the microsatellite dataset of charr in NewHybrids identified ten individuals as being admixed, i.e. almost 6% of all charr sampled, all from two different tributaries. One individual from Kobbhuvudbäcken was an F2 hybrid (*P* = 0.99), as were eight individuals from Stenbrottsbäcken (*P* = 0.95–0.99). There was also one individual from Stenbrottsbäcken that had equal probability (*P* = 0.50/0.50) of being a F2 hybrid or a backcross to Arctic charr. The individual from Kobbhuvudbäcken and two from Stenbrottsbäcken had Arctic charr mitochondrial haplotypes, i.e. their mother was Arctic charr. The remaining seven individuals had brook charr mitochondrial haplotypes and hence, mothers of brook charr origin.

### Population genetics and diversity

Summary tables of the tests of Hardy-Weinberg equilibrium (HWE) and the presence of null alleles are shown in Tables B, C, D in S1 File. One locus in Arctic charr, Sfo 23, consistently deviated from HWE and was removed from further analyses. All other tests revealed no consistent significant deviations from HWE. We observed evidence for potential null alleles in the data, most consistently in the brown trout locus Str 543, which was also removed from further analysis. In brook charr deviations from HWE and null alleles were observed in Stenbrottsbäcken, where hybridisation between the charr species was detected. One locus in Arctic charr, Sfo12, was monomorphic and removed from further analyses.

Microsatellite genetic diversity measures for each species are shown in Table 1. Overall values of diversity were moderate and quite similar among species and sampling locations within each species. Arctic charr were sampled from 2 tributaries (Stenbrottsbäcken not included as there were only 2 individuals and they were subsequently identified as hybrids) and 4 lake locations and had an overall corrected total heterozygosity (H’_T_) of 0.54 (*SD* 0.08), A_R_ of 4.93 (N_min_11) and G_IS_ of -0.063 (*SD* 0.089). Brook charr were sampled from 4 tributaries and had an H’_T_ of 0.60 (SD 0.10), A_R_ of 5.26 (N_min_14) and G_IS_ of 0.129 (*SD* 0.067). Finally, brown trout was sampled from 4 tributaries and 5 lake locations and had an H’_T_ of 0.66 (*SD* 0.06), A_R_ of 5.53 (N_min_10) and G_IS_ of 0.144 (*SD* 0.06). Inbreeding coefficients (G_IS_) in brook charr and brown trout were relatively high, particularly in tributary locations such as Stenbrottsbäcken (G_IS_ = 0.273), Herrån (G_IS_ = 0.223) and Myckelbäcken (G_IS_ = 0.174).

In Arctic charr overall F_ST_ was low but significant (F_ST_ = 0.01, *P* = 0.015, see Table E, F, G in S1 File for the full F_ST_ tables) suggesting only weak genetic structure. The only significant pairwise F_ST_ value (Bonferroni corrected *P* values < 0.008) was between Bunnerviken and Granön S, F_ST_ = 0.03. There was slightly higher genetic structure in brook charr among tributaries, with an overall F_ST_ = 0.04, *P* = 0.001. This was mainly driven by the differentiation among Klocka, Myckelbäcken and Stenbrottsbäcken. Overall F_ST_ in brown trout was also significant but low, F_ST_ = 0.02 (*P* = 0.001). Herrån was the most differentiated location with F_ST_ values from 0.04 to 0.11.

A high number of sibling relationships were detected in all three species (Tables 2, 3 and 4). Arctic charr had three full and 186 half sibling relationships, brown trout had 10 full and 376 half sibling relationships, and brook charr had 13 full and 320 half sibling relationships. These sibship pairs were found not only within sampling locations but also among most sampling locations across the lake and tributaries.

**Table 2:** Results of the COLONY analysis of sibling relationships for Arctic charr.

Location (N)	BN (5)	BV (20)	GN (12)	GS (21)	HD (22)	KB (3)
**Bunnerån (BN)**	0					
**Bunnerviken (BV)**	7	17				
**Granön N (GN)**	4	19	4 (1)			
**Granön S (GS)**	3	13	13	20 (1)		
**Handöl Delta (HD)**	5	18	10	23	11	
**Kobbhuvudb. (KB)**	0	3	2	3	1 (1)	0

N indicates sample size; values indicate the number of sibling relationships detected within and among sampling locations: half siblings outside brackets and full siblings inside brackets. Note: the sum of the sibling relationships detected can be larger than the sum of the sample sizes as individuals can have sibling relationships with more than one other individual.

**Table 3:** Results of the COLONY analysis of sibling relationships for brook charr.

Location (N)	KL (40)	KB (4)	MB (21)	SB (24)
**Klocka (KL)**	105 (5)			
**Kobbhuvudb. (KB)**	12	1		
**Myckelb. (MB)**	68 (2)	6	23 (2)	
**Stenbrottsb. (SB)**	30 (1)	7	27 (1)	36 (2)

N indicates sample size; values indicate the number of sibling relationships detected within and among sampling locations: half siblings outside brackets and full siblings inside brackets. Note: the sum of the sibling relationships detected can be larger than the sum of the sample sizes as individuals can have sibling relationships with more than one other individual.

**Table 4:** Results of the COLONY analysis of sibling relationships for brown trout.

Location (N)	AE (8)	BV (22)	GN (33)	GS (9)	HN (16)	KL (8)	KB (5)	MB (20)	VV (19)
**Årsön E (AE)**	2 (1)								
**Bunnerviken (BV)**	4	11							
**Granön N (GN)**	9	37 (2)	25 (2)						
**Granön S (GS)**	4	4 (1)	1	1					
**Herrån (HN)**	2	13	12	7	16 (1)				
**Klocka (KL)**	0	13	6	1	1	2			
**Kobbhuvudb. (KB)**	0	8	8	1	5	2	0		
**Myckelb. (MB)**	6	9	18	5	11	9	3	7	
**Västervik (VV)**	0	22	25 (1)	9	11 (1)	6 (1)	8	16	8

N indicates sample size; values indicate the number of sibling relationships detected within and among sampling locations: half siblings outside brackets and full siblings inside brackets. Note: the sum of the sibling relationships detected can be larger than the sum of the sample sizes as individuals can have sibling relationships with more than one other individual.

## Discussion

### Genetic diversity and inbreeding

The salmonid species of Lake Ånn had moderate levels of genetic diversity. Brown trout had the highest heterozygosity levels (H’_T_ = 0.66), followed by brook charr (H’_T_ = 0.60) and Arctic charr (H’_T_ = 0.54); the highest allelic richness values were also observed in brown trout (A_R_ = 5.53), followed by brook charr (A_R_ = 5.26) and Arctic charr (A_R_ = 4.93). These levels of diversity are similar among the three species, despite differences in population demographic history, particularly for brook charr, which were presumably founded from a small number of introduced fish. Although there was only one mtDNA haplotype detected in brook charr, suggesting a population bottleneck upon introduction, microsatellite diversity levels were comparable to the two native species, and may indicate that gene flow, potentially via introgression with the native Arctic charr (see below) or ongoing unreported introductions, has been a stronger factor than genetic drift in Lake Ånn.

In general, the levels of genetic diversity observed here are similar to previous population genetics studies on salmonids across their natural distribution. For brown trout: H_E_ = 0.44–0.71 in Sweden [66], H_E_ = 0.45–0.69 in Finland [67], H_E_ = 0.1–0.77 in Spain [68] and H_E_ = 0.73–0.81 in Switzerland [46]; Arctic charr: H_E_ = 0.72–0.87 in alpine European lakes [69] and H_O_ = 0.43–0.62 in wild Nordic populations [70]; brook charr: H_E_ = 0.60–0.69 in Canada [71–74]. Finally, a similar study to ours, assessing genetic diversity levels in three salmonids from a single drainage area in Canada [75, 76], also found comparable results: Arctic charr H_E_ = 0.705–0.765, brown trout H_E_ = 0.483–0.703, and brook trout H_E_ = 0.392–0.545. Thus, the levels of genetic diversity in the salmonids of Lake Ånn are within the range expected for these species, and from a conservation management perspective efforts should be made in order to maintain this standing genetic variation and evolutionary potential, e.g. continued environmental protection of the lake and monitoring of fishing activities.

Notably high levels of inbreeding (in this study measured as G_IS_ but is analogous to the more commonly used F_IS_) were observed in both brook charr (G_IS_ = 0.129) and brown trout (G_IS_ = 0.144) particularly in the tributaries. However, these levels of inbreeding are generally within the range of those observed in previous studies of these species (brown trout: F_IS_ up to 0.17, [66]; brook charr: F_IS_ up to 0.211, [74]; Arctic charr: F_IS_ up to 0.215, [71]), with the exception of brook charr in Stenbrottsbäcken (G_IS_ = 0.273) and brown trout in Herrån (G_IS_ = 0.223) and Årsön E (G_IS_ = 0.374), and likely reflect the fact that brook charr and brown trout spawn in tributaries and display spawning site fidelity. This means that related individuals or family groups return to the same restricted geographical location to reproduce each year, resulting in tributary locations that experience inbreeding and have lower effective population sizes and/or carrying capacities compared to lake locations. Indeed a high number of sibling relationships were detected across the entire dataset, most notably in brook charr, which is restricted to tributaries. It is well recognised that fish populations residing and/or reproducing predominantly in lakes versus streams have contrasting demographic dynamics, e.g. spawning and dispersal behaviour, with consequences for effective population sizes and genetic diversity [77]. Indeed, the effective population size of brook charr has been shown to vary over small geographic distances, driven by local habitat conditions [78]. Differences in effective population size have also been observed at the broader scale, e.g. marine vs freshwater fish in general [79, 80] as well as between sea migrating and resident trout [66]. Thus, our results may simply reflect this broad scale phenomenon at a local scale within a lake and its tributaries. Despite the high degree of inbreeding and kinship among individuals, genetic and allelic diversity was not alarmingly low and spatial genetic differentiation (overall F_ST_ = 0.01–0.04) indicated there is geneflow among spawning sites that is important for maintaining local genetic diversity. Although kinship associations may be responsible for elevating inbreeding coefficients and low effective population sizes, we recommend management measures to avoid the creation of even smaller and more isolated populations that in the long-term may pose genetic threats to overall population viability.

### Low genetic differentiation

Despite our expectation that genetic structure in Lake Ånn would be related to recognised spawning locations, there was evidence of only very low levels of genetic differentiation in all species across the study area. F_ST_ analyses suggested that there is only weak spatial differentiation: in the southern part of Lake Ånn for Arctic charr; of one tributary, Herrån, in brown trout; and among most tributaries in brook charr. This could be due to the small spatial scale of the study area, the dispersal behaviour of each species, and/or disruption of spawning sites due to introduced species or environmental changes.

Salmonids are renown for their high dispersal abilities, undergoing seasonal spawning migrations that may even require overcoming small waterfalls and weirs. In addition to the low F_ST_ values observed in Lake Ånn, kinship analyses detected numerous sibling pairs within and among sampling locations, indicating that fish disperse freely around the lake, and to some extent the tributaries. Thus, even if fish display spawning site fidelity (as indicated by those locations with significant although weak genetic differentiation), outside of the spawning season adults utilise all areas of the lake for foraging. Indeed, kin groupings in a native population of brook charr have also been found to be quite weak between foraging and spawning periods due to natural mortality [81]. Previous studies of salmonids have detected a range of different spatial genetic structuring (e.g. [45, 69, 82]) and it has been suggested that regional adaptation is likely stronger than local adaptation [73], perhaps also contributing the lack of micro-phylogeographic structure observed in Lake Ånn. In addition, studies of brook charr in the species native distribution have highlighted the importance of historical events on current genetic differentiation, i.e. drift following colonisation, with watersheds/basins being the predominant level of differentiation [74, 83]. Thus the small spatial scale of the study area coupled with the high dispersal abilities of salmonids could explain the lack of genetic structure observed in Lake Ånn.

In addition, the roles of introduced species and environmental change in shaping genetic structure needs to be considered. In the 1960s local fisherman mapped the location of known spawning locations of Arctic charr in and around Lake Ånn [53]. However, recent modifications in the lake environment due to the introduced lake trout, *Salvelinus namaycush*, and brook trout, as well as climate change have altered the previously observed spawning behaviour and grounds (P. Jämting pers. comm.). This problem has also been observed in Lake Windermere in the UK, where only 7 out of 12 spawning grounds of Arctic charr are still in use, because of eutrophication and sedimentation due to climate change and introduced species [84]. Thus it is possible that the genetic structure we observed is a reflection of past spawning assemblages that are gradually being broken down and admixed. Also, as the native brown trout and introduced brook charr have similar niche requirements and are forced into concurrency, their competitive interactions may influence habitat selection and subsequently genetic structure. For example, the introduction of brook charr may have disrupted previous spawning sites of brown trout and induced admixture. The importance of spawning and rearing habitat and absence of competitors and predators to overall production in salmonids is well recognised [85]. Further behavioural studies of spawning and dispersal behaviour of the salmonids in Lake Ånn may help provide further insights.

### Native and introduced charr hybridisation

Hybridisation between Arctic charr and brook charr was first described in a natural situation, where there was a large difference in population size, brook charr being rarer, with the resulting hybrids displaying many overlapping characteristics of morphology and behaviour [86]. This study found evidence for hybridisation between native populations of Arctic charr and the introduced brook charr, around 6% of all charr individuals were hybrids, with all hybrids being found in streams. The majority of hybrids were identified as F2 individuals with the maternal line (mtDNA) being contributed from both brook charr (seven individuals) and Arctic charr (three individuals). It is well recognised that it can be difficult to determine F2 individuals from further generation hybrids and/or backcrosses even with a large number of loci [25], thus as we used only eight loci in this analysis, the hybrids identified here could also be from further generations/backcrosses and the extent of hybridisation may have been underestimated.

The majority of hybrid individuals were sampled from Stenbrottsbäcken, a stream in the south-eastern part of the Lake Ånn drainage. This stream is in close proximity to several other larger streams where Arctic charr is known to undergo upstream spawning migrations. Thus the co-occurrence of the two species during the spawning period could facilitate hybridisation. If all hybrids are the result of stream-spawning parents, it could also explain why all hybrid individuals were sampled in, and seemingly prefer, stream locations. The majority of salmonids have polygynous mating systems, meaning that male fitness depends on the availability of females, perhaps leading to male biased dispersal in order to reduce competition [87]. In the case of hybrids from Stenbrottsbäcken in Lake Ånn, the majority of the maternal lineages were from brook charr, suggesting that it is Arctic charr males that are dispersing and competing for reproductive success, either by outcompeting brook charr males or engaging in sneaky behaviour. This implies that brook charr is having a negative impact on Arctic charr, by occupying valuable spawning grounds and leading to a loss of reproductive effort. A similar scenario has occurred in Japan, where introduced brook charr hybridise with native white-spotted charr, *Salvelinus leucomaenis*, and although introgression has not yet been detected, the negative impacts due to wasted reproductive resources are a recognised problem [88]. There may also be long-term consequences for the genetic integrity of both species, such as complete replacement of parts of the genome [50, 51], or loss of ‘native genetic variation’ if introgressed species are eradicated [19, 89]. Alternatively, the sharing of genes between the charr species may actually benefit the long-term survival of Arctic charr under climate change, as brook charr has a higher tolerance for warmer conditions (*sensu* [50, 51]). However, due to the widespread distribution of the introduced brook charr in Scandinavia and hence the widespread potential for hybridisation, further investigation of behaviour during spawning, monitoring of movements/tagging studies, determining the morphological differentiation and ecological niche of hybrids, as well as assessing whether there is any hybridisation with a second introduced species, lake trout *Salvelinus namaycush* (see [71]), would be valuable from a conservation perspective in order to further elucidate the impact of introduced salmonids on natives.

## Supporting Information

S1 File **Table A** Details of the primers and multiplexing used in this study. SA = *Salvelinus alpinus*, SF = *Salvelinus fontinalis*, ST = *Salmo trutta*. **Tables B, C, D** Results of the tests of Hardy-Weinberg equilibrium (HWE) and the presence of null alleles (NA) and stuttering (S) (Microchecker v2.2.3) in all three species. Locus and population combinations not in HWE (significant at P < 0.001 Bonferroni adjusted) are shown in bold. **Tables E, F, G** Pairwise F_ST_ tables for all three species. F_ST_ values in the lower triangle, *P* values in the upper triangle. Bonferroni corrected significant F_ST_ values shown in bold.
(DOC)

S1 Tables Spreadsheet containing the microsatellite genotype data generated in this study. (XLSX)
