# Supplementary material for: Genetic Diversity and Hybridisation between Native and Introduced Salmonidae Fishes in a Swedish Alpine Lake
Source: PLoS One. 2016 Mar 31;11(3):e0152732. doi: 10.1371/journal.pone.0152732 (PMC4816307; doi:10.1371/journal.pone.0152732)
Supplement: S1 File — Table A Details of the primers and multiplexing used in this study. SA = Salvelinus alpinus, SF = Salvelinus fontinalis, ST = Salmo trutta. Tables B, C, D Results of the tests of Hardy-Weinberg equilibrium (HWE) and the presence of null alleles (NA) and stuttering (S) (Microchecker v2.2.3) in all three species. Locus and population combinations not in HWE (significant at P < 0.001 Bonferroni adjusted) are shown in bold. Tables E, F, G Pairwise FST tables for all three species. FST values in the lower triangle, P values in the upper triangle. Bonferroni corrected significant FST values shown in bold. (DOC) [file pone.0152732.s001.doc]

**Table A Details of the primers and multiplexing used in this study.**

| Multiplex | Primer | Sequence | Species | Reference |
| --- | --- | --- | --- | --- |
| SMP1 | Ssa197 | F: GGGTTGAGTAGGGAGGCTTG  R: TGGCAGGGATTTGACATAAC | SA SF ST | 1 |
|  | Ssa403 | F: CTTTAGAAGACGGCTCACCCTGTA  R: GCTACTTCGTACTGACTGCCTCA | SA ST | 2 |
|  | Ssa408 | F: AATGGATTACGGGTACGTTAGACA  R: CTCTGTGCAGGTTCTTCATCTGT | ST | 2 |
|  | Ssa422 | F: TTATGGGCGTCCACCTCTGACA  R: CACCCCAGCCTCCTCAACCTTC | SA ST | 2 |
| SMP2 | Ssa406 | F: ACCAACCTGCACATGTCTTCTATG  R: GCTGCCGCCTGTTGTCTCTTT | SA | 2 |
|  | Sfo8 | F: CAACGAGCACAGAACAGG  R: CTTCCCCTGGAGAGGAAA | SA SF ST | 3 |
|  | SfoD75 | F: GTAGTGCCAAAACAGGTAGAGC  R: CATCCTTATTCCAACCTCAATC | SA SF ST | 4 |
| SMP3 | Pumf27 | F:TGCATGAGCGAGCGCATGAGTG  R: AAACCTCACCAACCTTCCCAC | SA SF ST | 5 |
|  | Str543 | F: CGGAATGCCATTTTTCACTC  R: GTTTGCCACGTTCTACAGTCAGC | SA ST | 6 |
| AMP1 | Sfo12 | F: GGTTTTGAAGAGTGACAG  R: CCCGTTTCACAATCAGAG | SA SF | 3 |
|  | Sfo23 | F: GTGTTCTTTTCTCAGCCC  R: AATGAGCGTTACGAGAGG | SA SF | 3 |
| AMP2 | Sfo18 | F: TGGTGTATCCTGCTCCTG  R: TGGAATGTGTGTCTGTTTTCT | SA SF | 3 |
|  | SfoC86 | F: ACCGATGGCCTTCAACAC  R: ATAGGCCCCTACCTCAAACC | SA SF | 4 |
|  | SfoC115 | F: CAGTTTCTATCTCCAGGCAATC  R: TTCTGAAAGCACTCAACATGG | SA | 4 |
|  | Ocl2 | F: ATTGACCGGTGAAACTCGAC  R: AACATACCCACACACACGGA | SA SF | 7 |
| TMP1 | Ssa417 | F: AGACAGGTCCAGACAAGCACTCA  R: ATCAAATCCACTGGGGTTATACTG | ST | 2 |
|  | Oneu9 | F: CTCTCTTTGGCTCGGGGAATGTT  R: GCATGTTCTGACAGCCTACAGCT | ST | 8 |
|  | Pumf69 | F: TRAAGGCAGAGTGTAAGAGC  R: GAGGACAGGCAGTCTTTATTGG | ST | 5 |

SA = *Salvelinus alpinus*, SF = *Salvelinus fontinalis*, ST = *Salmo trutta*.

References

1. O’Reilly P, Hamilton L, McConnell S, Wright J (1996) Rapid analysis of genetic variation in Atlantic salmon (Salmo salar) by PCR multiplexing of dinucleotide and tetranucleotide microsatellites. Can J Fish Aquat Sci 53: 2292-2298.

2. Cairney M, Taggart J, Høyheim B (2000) Characterization of microsatellite loci in Atlantic salmon (*Salmo salar* L.) and cross-species amplification in other salmonids. Mol Ecol 9: 2175-2178.

3. Angers B, Bernatchez L (1996) Usefulness of heterologous microsatellites obtained from brook charr, *Salvelinus fontinalis* Mitchill, in other *Salvelinus* species. Mol Ecol 5: 317-319.

4. King T, Lubinski B, Burnahm-Curtis M, Stott W, Morgan II R (2012) Tools for the management and conservation of genetic diversity in brook trout (*Salvelinus fontinalis*): tri- and tetranucleotide microsatellite markers for the assessment of genetic diversity, phylogeography, and historical demographics. Conservation Genet Resour 4: 539-543.

5. Kahilainen K, Teacher A, Kähkönen K, Vinni M, Lehtonen H, Merilä J (2011) First record of natural hybridization and introgression between pikeperch (*Sander lucioperca*) and perch (*Perca fluviatilis*). Ann Zool Fennici 48: 39-44.

6. Estoup A, Rousset F, Michalakis Y, Cornuet JM, Adriamanga M, Guyomard R (1998) Comparative analysis of microsatellite and allozyme markers: a case study investigating microgeographic differentiation in brown trout (*Salmo trutta*). Mol Ecol 7: 339-353.

7. Condrey M, Bentzen P (1998) Characterization of coastal cutthroat trout (Oncorhynchus clarki clarki) microsatellites and their conservation in other salmonids. Mol Ecol 7: 787-789.

8. Scribner K, Gust J, Fields R (2001) Isolation and characterization of novel salmon microsatellite loci: cross-species amplification and population genetic applications. Can J Fish Aquat Sci 53: 833-841.

**Table B Results of the tests of Hardy-Weinberg equilibrium (HWE) and the presence of null alleles (NA) and stuttering (S) in *Salvelinus alpinus*.**

|  | Bunnerån | Bunnerviken | Granön N | Granön S | Handöl Delta |
| --- | --- | --- | --- | --- | --- |
| Ocl 12 |  |  |  |  |  |
| Pumf 27 |  |  |  |  |  |
| Sfo 8 |  |  |  |  |  |
| Sfo 12 |  |  |  |  |  |
| Sfo 18 |  |  |  |  |  |
| Sfo 23 |  | **xHWE** NA | **xHWE** NA | **xHWE** NA | **xHWE** NA |
| Sfo C86 |  |  |  |  |  |
| Sfo C115 |  |  |  |  |  |
| Sfo D75 |  | NA |  |  |  |
| Ssa 197 |  |  | NA |  |  |
| Ssa 403 |  |  |  |  |  |
| Ssa 406 |  | NA |  |  | NA, S |
| Ssa 422 |  |  |  |  |  |
| Str 543 |  |  |  |  |  |

Locus and population combinations not in HWE (significant at P < 0.001 Bonferroni adjusted) are shown in bold.

**Table C Results of the tests of Hardy-Weinberg equilibrium (HWE) and the presence of null alleles (NA) and stuttering (S) in *Salvelinus fontinalis*.**

|  | Klocka | Kobbhuvudb. | Myckleb. | Stenbrottsb. |
| --- | --- | --- | --- | --- |
| Ocl 12 |  |  |  |  |
| Pumf 27 |  |  | NA | NA |
| Sfo 8 |  |  |  | **xHWE** NA |
| Sfo 12 |  |  |  | **XHWE** NA, S |
| Sfo 18 |  |  | NA | NA |
| Sfo 23 |  |  |  | NA |
| Sfo C86 |  |  |  |  |
| Sfo D75 |  |  |  |  |
| Ssa 197 |  |  |  |  |

Locus and population combinations not in HWE (significant at P < 0.001 Bonferroni adjusted) are shown in bold.

**Table D Results of the tests of Hardy-Weinberg equilibrium (HWE) and the presence of null alleles (NA) and stuttering (S) in *Salmo trutta*.**

|  | Bunner-  viken | Granön N | Granön S | Herrån | Klocka | Kobb-  huvudb. | Myckelb. | Väster-  vik |
| --- | --- | --- | --- | --- | --- | --- | --- | --- |
| Oneu 9 | NA | NA |  |  |  |  |  |  |
| Pumf 27 |  |  |  |  |  |  |  |  |
| Pumf 69 |  | NA |  |  |  |  |  |  |
| Sfo 8 | NA |  |  |  |  |  |  |  |
| Sfo D75 | NA | **xHWE** NA, S |  | NA |  |  |  |  |
| Ssa 197 |  |  |  |  |  |  |  |  |
| Ssa 403 |  |  |  |  |  |  | NA |  |
| Ssa 408 |  |  |  |  |  |  | NA |  |
| Ssa 417 |  | NA |  |  |  |  |  | NA |
| Ssa 422 | NA, S |  |  |  |  |  |  | NA |
| Str 543 | NA | NA | NA |  | NA |  | NA |  |

Locus and population combinations not in HWE (significant at P < 0.001 Bonferroni adjusted) are shown in bold.

**Table E Pairwise F_ST_ tables for *Salvelinus alpinus*.**

|  | Bunner-  viken | Granön  N | Granön  S | Handöl  Delta |
| --- | --- | --- | --- | --- |
| Bunnerviken |  | 0.210 | 0.001 | 0.009 |
| Granön N | 0.008 |  | 0.191 | 0.186 |
| Granön S | **0.031** | 0.008 |  | 0.351 |
| Handöl Delta | 0.017 | 0.008 | 0.002 |  |

F_ST_ values in the lower triangle, *P* values in the upper triangle. Bonferroni corrected significant F_ST_ values shown in bold.

**Table F Pairwise F_ST_ tables for *Salvelinus fontinalis.***

|  | Klocka | Kobb-  huvudb. | Myckleb. | Sten-  brottsb. |
| --- | --- | --- | --- | --- |
| Klocka |  | 0.165 | 0.001 | 0.001 |
| Kobbhuvudb. | 0.020 |  | 0.614 | 0.762 |
| Myckleb. | **0.050** | -0.012 |  | 0.001 |
| Stenbrottsb. | **0.060** | 0.040 | **0.040** |  |

F_ST_ values in the lower triangle, *P* values in the upper triangle. Bonferroni corrected significant F_ST_ values shown in bold.

**Table G Pairwise F_ST_ tables for *Salmo trutta.***

|  | Årsön  E | Bunner-  viken | Granön  N | Granön  S | Herrån | Klocka | Kobb-  huvudb. | Myckelb. | Väster-  vik |
| --- | --- | --- | --- | --- | --- | --- | --- | --- | --- |
| Årsön E |  | 0.022 | 0.246 | 0.758 | 0.002 | 0.030 | 0.244 | 0.304 | 0.015 |
| Bunnerviken | 0.031 |  | 0.587 | 0.085 | 0.001 | 0.033 | 0.735 | 0.071 | 0.197 |
| Granön N | 0.008 | -0.002 |  | 0.133 | 0.001 | 0.002 | 0.124 | 0.063 | 0.125 |
| Granön S | -0.017 | 0.021 | 0.014 |  | 0.015 | 0.022 | 0.554 | 0.325 | 0.315 |
| Herrån | 0.068 | **0.059** | **0.053** | 0.044 |  | 0.002 | 0.032 | 0.001 | 0.002 |
| Klocka | 0.058 | 0.033 | 0.067 | 0.053 | 0.105 |  | 0.674 | 0.040 | 0.001 |
| Kobbhuvudb. | 0.015 | -0.013 | 0.023 | -0.007 | 0.050 | -0.019 |  | 0.488 | 0.419 |
| Myckelb. | 0.006 | 0.012 | 0.011 | 0.004 | **0.047** | 0.031 | -0.001 |  | 0.406 |
| Västervik | 0.043 | 0.007 | 0.008 | 0.005 | 0.042 | **0.062** | 0.001 | 0.001 |  |

F_ST_ values in the lower triangle, *P* values in the upper triangle. Bonferroni corrected significant F_ST_ values shown in bold.
